# First Description of KPC-2-Producing *Klebsiella oxytoca* Isolated from a Pediatric Patient with Nosocomial Pneumonia in Venezuela

**DOI:** 10.1155/2014/434987

**Published:** 2014-10-22

**Authors:** Indira Labrador, María Araque

**Affiliations:** Laboratorio de Microbiología Molecular, Departamento de Microbiología y Parasitología, Facultad de Farmacia y Bioanálisis, Universidad de Los Andes, Mérida 5101, Venezuela

## Abstract

During the last decade, carbapenem resistance has emerged among clinical isolates of the Enterobacteriaceae family. This has been increasingly attributed to the production of *β*-lactamases capable of hydrolyzing carbapenems. Among these enzymes, *Klebsiella pneumoniae* carbapenemases (KPCs) are the most frequently and clinically significant class-A carbapenemases. In this report, we describe the first nosocomial KPC-2-producing *K. oxytoca* isolated from a pediatric patient with pneumonia admitted to the intensive care unit at The Andes University Hospital, Mérida, Venezuela. This strain was resistant to several antibiotics including imipenem, ertapenem, and meropenem but remained susceptible to ciprofloxacin, colistin, and tigecycline. Conjugation assays demonstrated the transferability of all resistance determinants, except aminoglycosides. The isolate LMM-SA26 carried a ~21 kb conjugative plasmid that harbored the *bla*_KPC-2_, *bla*_CTX-M-8_, and *bla*_TEM-15_ genes. Although carbapenem resistance in the Enterobacteriaceae is still unusual in Venezuela, KPCs have a great potential to spread due to their localization on mobile genetic elements. Therefore, rapid detection of KPC-carrying bacteria with phenotypic and confirmatory molecular tests is essential to establish therapeutic options and effective control measures.

## 1. Introduction

Carbapenems have been considered as first-line therapy for treatment of severe infections caused by multidrug-resistant (MDR) Gram-negative bacteria, especially strains producing high levels of AmpC cephalosporinases or extended spectrum *β*-lactamases (ESBLs). Unfortunately, the extensive use of carbapenems has resulted in the emergence of resistant Enterobacteriaceae strains. This resistance is the major threat for treatment of these infections and production of carbapenemases is the most important molecular mechanism, from both epidemiologic and clinical standpoints [1]. These enzymes in Enterobacteriaceae are represented by three molecular classes of *β*-lactamases: A, B, and D. KPC is a molecular class-A serine *β*-lactamase belonging to the functional group 2f, which is capable of hydrolyzing carbapenems, penicillins, cephalosporins, and aztreonam, and is weakly inhibited by clavulanic acid and tazobactam [2]. KPCs were first detected in 2001 in North Carolina (USA) [3] and since then have spread all over the United States [4, 5] and other countries worldwide [6]. *bla*_KPC_ have been identified among Enterobacteriaceae isolates and recently in* Pseudomonas aeruginosa* and* Acinetobacter baumannii* [2]. The genes encoding KPC-1 and KPC-2 were found to be identical. Hence, ten KPC variants (KPC-1/2 to KPC-11) have been described so far (http://www.lahey.org/studies/) differing in 1- or 2-point mutations [5]. These carbapenemases-encoding genes are found on transferable plasmids mainly associated with transposon Tn*4401* [4]. Furthermore, plasmids carrying *bla*_KPC_ are often also linked to resistance determinants for other antibiotics, which lead to multidrug-resistance (MDR) events [5]. KPC-producing isolates may be difficult to detect since high carbapenem MICs are not always evident. This poses a major therapeutic challenge, as treatment may be inadequate and therefore this may lead to a significant increase in patients' mortality, morbidity, and hospitalization costs [1].

Few reports describe the presence of KPC in* Klebsiella oxytoca* [7, 8], since KPC enzymes are predominantly found in* K. pneumoniae* [5]. Recently, Almeida et al. identified the first nosocomial KPC-2-producing* K. oxytoca* strain in Brazil [9] and, to our knowledge, only one outbreak caused by KPC-producing* K. oxytoca* has been described so far [10]. In this report we describe the first case of KPC-2-producing* K. oxytoca* isolated in a pediatric patient with nosocomial pneumonia from Venezuela. This strain also coproduced other *β*-lactamases as CTX-M-8 and TEM-15.

## 2. Case Presentation

A 3-year-old child with severe respiratory distress was admitted to the pediatric emergency (PE) of The Andes University Hospital, Mérida, Venezuela, in September 2013. Ten days before his admission he was hospitalized for complicated bronchiolitis, which was treated with a course of intravenous cefoperazone and gentamicin. The patient's past medical history was notable for frequent episodes of reactive airway disease, treated with bronchodilators and short courses of corticosteroids. At presentation, the child was in bad general conditions revealing mucocutaneous paleness and tachycardic, lethargic, and severe respiratory distress, which merited immediate intubation and ventilation assistance. On admission, a chest radiograph revealed a diffuse interstitial pattern. The patient was transferred to the intensive care unit (ICU) and started on empirical cefotaxime (100 mg/kg/day) and clarithromycin (15 mg/kg/day) for presumed interstitial pneumonia. Tracheal secretion aspirate cultures were negative. However, after 3 days of hospitalization in the ICU with assisted ventilation, the patient's clinical condition worsened. Blood samples were collected for culture and antibiotics were changed to meropenem (60 mg/kg/day). A new chest X-ray reported upper right and lower lobe opacities and a small right pleural effusion was detected by a tomography scan. Two days later, blood cultures reported the presence of a carbapenem-resistant* K. oxytoca* (LMM-SA26). Epidemiological analysis of patient and his immediate family members did not reveal any recent travel to settings of KPC endemicity. The pleural effusion was resolved without drainage, and the patient recovered well and was discharged from the hospital after 10 days of treatment with colistin (5 mg/kg/day) plus ciprofloxacin (30 mg/kg/day).

The strain was recovered in BacT/ALERT 3D 60 (BioMérieux, Marcy l'Etoile, France) culture vials and subcultured on 5% of sheep blood agar (BBL, Becton Dickinson, Cockeysville, MD, USA) and MacConkey agar (BBL). Gram stains revealed the presence of Gram-negative bacilli. Bacterial identification was carried out by matrix-assisted laser desorption ionization-time of flight (MALDI-TOF) mass spectrometry (VITEK MS MALDI-TOF, BioMérieux) which reported* K. oxytoca* (99.9% score identifier). The antimicrobial susceptibility profile was determined by the broth dilution method according to the Clinical and Laboratory Standards Institute (CLSI) guidelines [11]. LMM-SA26 isolate showed resistance to broad-spectrum cephalosporins (MIC; 8–>256 *μ*g/liter), carbapenems (MIC; 8–>64 *μ*g/liter), and aminoglycosides (MIC; 16–>64 *μ*g/liter) but remained susceptible to nalidixic acid, ciprofloxacin, colistin, and tigecycline (Table 1). The ESBL and carbapenemase activity were confirmed by the double disk (Oxoid, Basingstoke, Hampshire, England) synergy test (DDST) and modified Hodge test (MHT), respectively [12]. Also, we included a disk diffusion assay using ertapenem and 3-aminophenyl boronic acid (400 *μ*g) as an additional confirmatory phenotypic test of activity of KPC-type *β*-lactamase [13]. The presence of a metallo-*β*-lactamases was discarded because of a negative result of the synergism test using imipenem and EDTA disks according to CLSI [12].

To determine whether the resistance was transferable, conjugation experiments using mixed broth cultures were performed with* E. coli* MKD135 (*argH, rpoB18, rpoB19, recA, rpsL*) as recipient. Transconjugants were selected on Mueller Hinton agar (BBL) plates containing rifampicin (400 mg/liter) and cefotaxime (2 mg/liter). All resistance determinants were successfully transferred to* E. coli* MKD135, except aminoglycoside resistance (Table 1). Plasmid DNA from transconjugants was extracted by the rapid alkaline lyses method [14].

The presence of *bla*_TEM_, *bla*_CTX-M_, *bla*_CTX-M-8_, and *bla*_KPC_ was determined by PCR using specific primers and conditions previously described [15, 16]. Amplification products were purified (PCR-Accuprep kit Bioneer) and their nucleotide sequencing was performed with the ABI Prism 377 genetic analyzer (Applied Biosystems, CA, USA). Nucleotide and amino acid sequence alignments were analyzed using the Basic Local Alignment Search Tool (BLAST) suite of programs (http://www.ncbi.nlm.nih.gov/blast/Blast.cgi). This revealed the presence of the *bla*_TEM-15_, *bla*_CTX-M-8_, and *bla*_KPC-2_ genes, all with a 99% degree of identity, corresponding to the nucleotide sequence reference GenBank accession N° HQ877605, AF189721, and AY034847, respectively. PCR screening for other *β*-lactamase genes gave negative results.

## 3. Discussion

Over the last decade, multiresistant* K. pneumoniae* producing ESBLs causing outbreaks of nosocomial infection in The Andes University Hospital, Mérida, Venezuela, has been reported [17, 18]. These outbreaks have been characterized by a complex epidemiological situation, in which the presence of different clones and ESBLs has been involved [18, 19]. Therefore, these infections have been treated mainly with carbapenems. However, no molecular epidemiological data were available on the carbapenemase-producing Gram-negative bacteria in this hospital. In the present study, we report a nosocomial KPC-2-producing* K. oxytoca* strain that also coproduced *bla*_CTX-M-8_ and *bla*_TEM-15_ isolated from a pediatric patient with pneumonia hospitalized in the ICU of The Andes University Hospital. This strain displayed an unusual multidrug resistance pattern that dramatically limited the possible therapeutic options [20, 21]. Indeed, the child was treated with a combination of colistin and ciprofloxacin, following failure of an empirical treatment based in cephalosporins plus clarithromycin and carbapenems.

Cells transconjugants showed only resistance or reduced susceptibility to all *β*-lactams compared with the clinical strain (Table 1), suggesting that additional resistance mechanisms may contribute to the high-level resistance as well as resistance to other non-*β*-lactam antibiotics such as aminoglycosides. Although not determined in this study, alterations in porin expression are known to affect MICs of these antibiotics in* Klebsiella* [22]. The *bla*_KPC_ genes have usually been identified in self-transferable plasmids varying in size and structure and frequently also carrying other *β*-lactamase genes such as ESBLs [5]. In our case,* K. oxytoca* LMM-SA26 harbored a conjugative plasmid of ~21 kb (pLMM-SA26) carrying genes encoding *bla*_KPC-2_, *bla*_CTX-M-8_, and *bla*_TEM-15_. Yigit et al. [7] reported the KPC-2 determinant in a MDR* K. oxytoca* strain localized on a 70 kb conjugative plasmid that also encoded SHV-26 and TEM-1. Similarly, Li et al. [8] described the coproduction of plasmid-mediated *bla*_KPC-2_ and *bla*_IMP-8_ genes in a fecal* K. oxytoca* strain isolated from a 5-year-old patient with acute leukemia.

Previous studies have shown that pediatric patients, with repeated and prolonged hospitalizations, use of broad-spectrum antibiotics, invasive procedures, particular intubation and ventilation assistance, and intravascular arterial or venous catheterization, are highly prone to acquire infections by multidrug-resistant bacteria [10, 20]. This is a possible scenario applicable to our case, besides previous hospitalizations history and use of steroids; the patient had an endotracheal tube and mechanical ventilation to treat the severe respiratory distress, indwelling vascular devices used for parenteral nutrition, and administration of broad-spectrum antibiotics.

Although the mechanism of transmission of KPC-2-producing* K. oxytoca* LMM-SA26 was not assessed, we speculate that a nosocomial acquisition through indirect patient contact occurred, possibly via transient hand colonization of personnel. Many studies have linked hands of health-care workers with the emergence of MDR* K. oxytoca* infections outbreaks [1–8, 19]. Therefore, the importance of hand washing and compliance with guidelines for preventing nosocomial infections were emphasized to the personnel at the time this case was studied. We stress the importance of an infection control program, based on active surveillance, and also of strict adherence to hand disinfection and the use of gloves.

Carbapenem resistance in the Enterobacteriaceae is still unusual in Venezuela [23] and, to the best of our knowledge, LMM-SA26 is the first nosocomial KPC-2-producing* K. oxytoca* isolated from a pediatric patient with pneumonia in this country. Additionally, our KPC-producing isolate was found to accumulate other *β*-lactam resistance enzymes as CTX-M-8 and TEM-15. These findings highlight that the emergence of the Enterobacteriaceae strains producing KPC associated to other resistance determinants may have serious consequences for clinical therapy. Rapid detection of KPC-carrying bacteria and ESBLs with phenotypic and confirmatory molecular tests is essential to establish therapeutic options and effective control measures.

## Figures and Tables

**Table 1:** Antibiotic susceptibility patterns of *K. oxytoca* LMM-SA26, *E. coli* LMM-SA26Tc transconjugant, and* E. coli* MKD135 recipient.

Antibiotics tested	MIC (mg/liter) for		
	*K. oxytoca* LMM-SA26	*E. coli* LMM-SA26Tc	*E. coli* MKD135
Cefotaxime	32	16	0.064
Cefotaxime/clavulanic acid^a^	8	4	0.125
Ceftazidime	>256	125	0.025
Ceftazidime/clavulanic acid^a^	32	8	0.25
Aztreonam	8	4	0.25
Imipenem	8	4	0.064
Ertapenem	>64	16	0.032
Meropenem	16	16	0.064
Gentamicin	>64	2	0.5
Amikacin	64	2	0.5
Tobramycin	16	1	0.125
Nalidixic Acid	1	0.064	1
Ciprofloxacin	2	0.125	0.25
Colistin	1	0.25	0.25
Tigecycline	0.25	0.125	0.125
Others test			
DDST	+	+	−
MHT	+	+	−
Ertapenem + boronic acid^b^	+	+	−
Imipenem + EDTA^c^	−	−	−

^a^Clavulanate was used at a fixed concentration of 4 mg/liter. ^b^The test was considered positive when boronic acid exhibited inhibitory effects increasing the bacterial growth-inhibitory zone to ≥5 mm around the ertapenem disk. ^c^The test was considered negative when EDTA did not increase the bacterial growth-inhibitory zone (≥5 mm) around the imipenem disk. DDST: double disk synergy test; MHT: modified Hodge test.
